# Association of leisure activity changes and reversion from mild cognitive impairment to normal cognitive function among older adults: A prospective cohort study

**DOI:** 10.3389/fpubh.2022.1035762

**Published:** 2022-11-22

**Authors:** Xin Yi Xu, Shan Shan Wang, Li Niu, Isaac Sze Him Leung, Qing Bao Tian

**Affiliations:** ^1^Postdoctoral Research Station in Basic Medicine, Hebei Medical University, Shijiazhuang, China; ^2^School of Nursing, Centre for Gerontological Nursing, The Hong Kong Polytechnic University, Hong Kong, Hong Kong SAR, China; ^3^School of Nursing and Health, Zhengzhou University, Zhengzhou, China; ^4^International Education College, Hebei Medical University, Shijiazhuang, China; ^5^Department of Statistics, The Chinese University of Hong Kong, Hong Kong, Hong Kong SAR, China; ^6^Department of Epidemiology and Statistics, School of Public Health, Hebei Medical University, Shijiazhuang, China

**Keywords:** leisure activity, mild cognitive impairment, reversion, older adults, longitudinal study

## Abstract

**Background:**

Older adults with mild cognitive impairment (MCI) have the possibility of reverting to normal cognitive function. Leisure activity engagement (LAE) plays a critical role in the progress of the cognitive function. A better understanding of the dynamic relationship between LAE and MCI reversion would inform the implementation of preclinical dementia interventions. This study aimed to investigate the association between change patterns of LAE and MCI reversion among older adults using the Chinese Longitudinal Healthy Longevity Survey (CLHLS) database.

**Study design:**

Longitudinal population-based study.

**Methods:**

Older adults with MCI at the baseline were enrolled in this study. Information about cognitive function, overall, cognitively stimulating, physically active/demanding, and socially engaged LAE was collected at baseline and follow-up. Adjusted hazard ratios (HRs) for reversion and 95% confidence intervals (CIs) were calculated by Cox hazard models with time as the underlying time metric. We also assessed potential effect modifications by creating a cross-product of the stratifying variable with LAE change patterns in the fully adjusted model.

**Results:**

The restricted cubic spline showed that the association between LAE change scores and MCI reversion rate was statistically significant and nonlinear (*p*<0.01). Taking participants in the low–low group as a reference, participants in the low–medium, low–high, medium–medium, medium–high, high–medium, and high–high groups had increased possibilities of MCI reversion with HRs (95% CI) of 2.19 (1.57–3.06), 2.97 (2.13–4.13), 0.87 (0.64–1.19), 2.28 (1.71–3.03), 2.78 (2.10–3.69), 1.93 (1.43–2.59), and 2.74 (2.09–3.60), respectively. Further stratified models showed that the impact of LAE change patterns on MCI reversion varied in different ages (nonagenarian, octogenarian, and younger elderly) and gender.

**Conclusions:**

Participants who maintained the highest LAE had the greatest possibility of MCI reversion. Meanwhile, a higher level of LAE maintenance was associated with the increased possibility of MCI reversion. These results provide a practical message to older adults about how dynamic changes in LAE are associated with improved cognitive function.

## Introduction

Mild cognitive impairment (MCI) is the transitional stage between normal aging and dementia in which individuals demonstrate objective cognitive impairment that does not interfere with daily functional independence (1). Recent evidence reported that the prevalence of MCI was around 14.8% in people aged 75–79 years and 25.2% in people aged 80–84 years (2). Even though older adults with MCI have a higher risk of progressing to dementia than individuals with normal cognitive function (3, 4), nearly 30% of them could still revert to normal cognitive function (5).

To date, few studies have focused on individuals who reverted to normal cognitive function after a diagnosis of MCI. Knowing the predictors of MCI reversion in older adults with the increasing need to treat dementia at an early stage is important. In addition, most previous studies investigated the association between MCI reversion and non-modifiable factors and lifelong factors that cannot be changed in later life (e.g., gender, age, educational level, economic status, and living place) (6, 7). Evidence on modifiable predictors for MCI reversion is still limited.

Leisure activity engagement (LAE) plays a critical role in the progress of cognitive function (8). Many resources found that LAE may delay or prevent Alzheimer's disease (AD) or related dementia (9). However, few researchers studied the impact of LAE on MCI reversion. It is critical to investigate the impact of LAE on MCI reversion among community-dwelling older adults to prevent dementia at an early stage. It is also valuable to differentiate the types of leisure activities, that is, cognitive, physical, and social when studying their effects on MCI reversion since these three subtypes of LAE have varied pathways which converge within three major etiological hypotheses (such as the cognitive reserve hypothesis, the vascular hypothesis, and the stress hypothesis) for dementia and other cognitive disorders (10).

Although a cohort study identified some specific activities positively associated with MCI reversion (4), the LAE and cognitive ability measurements were conducted at a given age. They only assessed the change in cognitive ability from the point award, which ignored dynamic features of LAE over time and could introduce some measurement errors (11, 12). It is critical to capture changes in LAE since they reflect the associated risks when individuals change their lifestyle in the real world from a public health perspective (13). A better understanding of the dynamic relationship between LAE and MCI reversion would inform the implementation of preclinical dementia interventions and improve our understanding of the aging mind and the brain.

The association between changes in LAE, such as changing from a higher engagement at the baseline to a lower engagement at follow-up, and MCI reversion rate remains unclear, and we aimed to fill in this blank. To address this question, we investigated the association between change patterns of overall, cognitive-based, physical-based, and social-based LAE and MCI reversion among older adults by using the data from the Chinese Longitudinal Healthy Longevity Survey (CLHLS) database.

## Materials and methods

### Study design, participants, and procedures

Participants were selected from older adults enrolled in the population-based cohort study titled CLHLS. The CLHLS was a nationwide prospective cohort study that enrolled individuals aged 65 years or older. The sample was randomly selected from 806 cities and counties in 23 provinces of China using multi-stage stratified sampling, covering about half of the cities and counties in each province (14). More detailed information on the study design and data quality assessment of the CLHLS has been presented in previous studies (15). All baseline and follow-up surveys were administered at the homes of participants by trained interviewers with a structured questionnaire. Proxy respondents, usually a spouse or other close family members, were interviewed when the participants were unable to answer questions, but questions regarding cognitive function were answered by the participants themselves.

**Figure 1:**
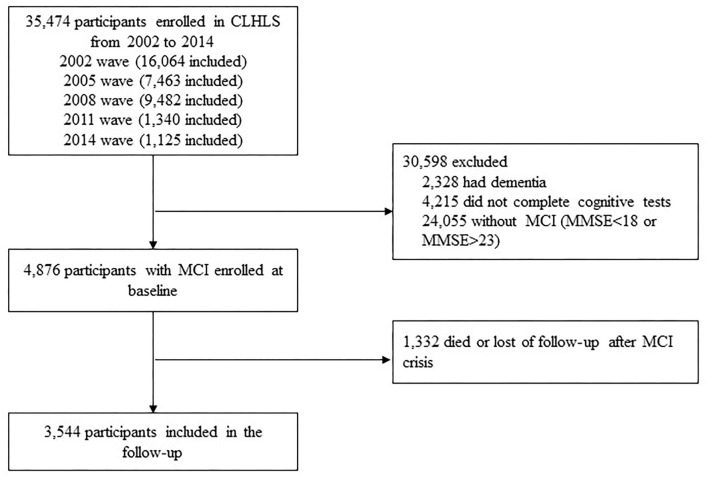
Flow diagram of sample selection.

In this study, we included participants who had MCI at the baseline. The baseline exclusion criteria were health problems, such as clinical diagnosis of dementia, missing data regarding the exclusion criteria, relocation or death during the follow-up period, without MCI (MMSE<18 or MMSE>23) at the baseline. Among 35,474 participants enrolled in the CLHLS from 2002 to 2014, 31,930 participants were excluded according to the exclusion criteria. Finally, 3,544 participants with MCI at the baseline were enrolled in the study (Figure 1).

The Chinese Longitudinal Healthy Longevity Survey study was approved by the Institutional Review Board of Duke University (Pro00062871) and the Biomedical Ethics Committee of Peking University (IRB00001052-13074). Each participant signed written informed consent.

### Assessment of leisure activity

The frequency of engaging in eight typical leisure activities: housework, personal outdoor activities, garden work, reading newspapers/books, raising domestic animals, playing cards and/or Mahjong, watching TV and/or listening to the radio, social activities (organized) was recorded as “almost every day,” “not daily, but once a week,” “not weekly, but at least once a month,” “not monthly, but sometimes,” or “never.” The frequency of engaging in each activity was recoded on a three-point Likert scale: frequently, occasionally, and rarely, and they were scored as 2, 1, and 0, respectively. The classification resembles that used in a study by Fernández-Mayoralas, Rojo-Pérez (16), which categorized participation in leisure activities as “active,” “moderately active,” and “inactive.” This categorization has drawn out more differences among the three categories, as opposed to categorizing participation into a two-category variable (“active” and “inactive”).

According to the predominant element of each activity, these activities were categorized into cognitively stimulating, physically active/demanding, and socially engaged (17). The range of overall, cognitively stimulating (such as reading newspapers/books and watching TV/listening to the radio), physically active/demanding (such as housework, personal outdoor activities, and garden work), and socially engaged (such as playing cards/Mahjong, engagement in social activities, and raise domestic animals) were 0–16, 0–4, 0–6, and 0–6, respectively, following established studies (18).

### Definitions of LAE change patterns

Leisure activity engagement (LAE) change patterns of overall, cognitively stimulating, physically active/demanding, and socially engaged were calculated according to LAE from the baseline to follow-up. The overall LAE was categorized into three groups [low (0–2 score), medium (3–5 score), and high (6–16 score] according to the distribution of participants. Then, nine relative LAE change patterns were created: low–low, low–medium, low–high, medium–low, medium –medium, medium–high, high–low, high–medium, and high–high. Accordingly, cognitively stimulating LAE was categorized into low, medium, and high with 0, 1–2, and 3-−4, and physically active/demanding LAE was categorized into three groups with 0, 1–3, and 4–6, and socially engaged LAE was categorized into three groups with 0, 1, and 2–6.

### Cognitive assessment

The mild cognitive impairment was measured by the Chinese version of the Mini-Mental State Examination (MMSE) and adapted and validated from the scale developed by Folstein et al. (19). The Chinese version of the MMSE considers the cultural and socioeconomic conditions of the older Chinese adults so that all question items in the test could be easily comprehended and answered by a survey participant with normal cognitive functioning (20). The Chinese version of the Mini-Mental State Examination (CMMSE) made small modifications based on the social-cultural differences of the Chinese population. According to regional divisions of China, test items for the orientation to the place were adapted as to province, district, street, place, and floor to replace the phrase country, town, street, place, and floor. The reliability and validity of the CMMSE in the CLHLS have been established in prior studies (21, 22). In particular, previous research showed that participants were more likely to be unable to answer relatively difficult tasks when they exhibited poor health and/or existing cognitive limitations (23). Therefore, following prior research, we categorized responses of “unable to answer” as incorrect answers. This approach has been widely used in previous studies and will not introduce potential bias (24). The MMSE measured 5 aspects of cognitive function (such as orientation, reaction, attention and calculation, recall, and language) by 24 items. The total score ranged from 0 to 30 and a higher score indicated better cognitive function. Mild cognitive impairment (MCI) was defined as a CMMSE score between 18 and 23 (25, 26), and reversion was defined as a participant with MCI reaching 24 or above in the follow-up tests for the first time. Reliable change in MMSE should be at least 2 to 4 points (27), and we added an additional restriction of MMSE change≥3 points to the definition of MCI reversion. Sensitivity analysis was also conducted for other thresholds (≥4).

### Covariates

The following covariates were assessed: age, sex, residence (urban or rural), education (years of schooling), marital status (married, divorced/widowed/never), economic status (favorable or unfavorable), living pattern (living with family members, alone, or at the nursing home), number of people living with, smoke at present (yes or no), drinking alcohol at present (yes or no), exercise at present (yes or no), activity of daily living (ADL), instrumental ADL (IADL), and chronic diseases including hypertension, diabetes, heart disease, stroke or cardiovascular disease (CVD), cataract, digestive system diseases, arthritis, and Parkinson's disease were coded into yes and no.

The activity of daily living (ADL) was measured at each wave using six items (such as dressing, bathing, indoor transferring, toileting, continence, and feeding). Participants were asked if they needed assistance with each of the six activities. Instrumental ADL (IADL) was composed of eight items (such as shopping, visiting neighbors, washing clothes, making food, walking 1 km, crouching and standing [repeated three times], carrying 5 kg weight, and taking public transport). Respondents were categorized as having an IADL disability if they needed help in performing at least one of the eight items according to the Lawton scale (28).

### Statistical analysis

Baseline characteristics are presented as the mean (standard deviation [SD]) for continuous variables and the number (percentages [%]) for categorical variables. Pearson's Chi-squared test and *t*-test were used to examine the difference in the baseline characteristics of participants in different LAE change patterns. The reversion rate was calculated during the follow-up assessments. We applied Cox hazard models with time as the underlying time metric to calculate the hazard ratios (HRs) and 95% confidence intervals (95% CIs) for analyzing the association between LAE changes and MCI reversion. We examined the proportional hazards assumption by creating a cross-product of follow-up time and LAE change patterns. Possible nonlinear relationships by nonparametrically restricted cubic splines were analyzed between the continuous LAE change points and MCI reversion (29, 30).

Demographic variables, functional ability, and chronic medical illness were listed as possible covariates. The association between changes in LAE and MCI reversion was investigated in three models: Model 1, adjusted for sex and age; Model 2, further adjusted for residence, years of schooling, marital status, economic status, living pattern, and the number of people living with based on Model 1; Model 3, further adjusted for smoking, alcohol drinking, ADL, IADL, and chronic diseases (such as hypertension, diabetes, heart disease, stroke or CVD, cataract, digestive system diseases, arthritis, and Parkinson's disease) based on Model 2. Adjusted hazard ratios (HRs) for reversion and 95% confidence intervals (CIs) were calculated.

We performed stratified analyses to evaluate potential effect modifications by baseline age (younger elderly 65–79 years, octogenarian 80–89 years, nonagenarian: ≥90 years), sex (male or female), residence (urban or rural), marital status (married, divorced/widowed/never), economic status (favorable or unfavorable), living pattern (living with family members, alone, or at the nursing home). We assessed potential effect modifications by creating a cross-product of the stratifying variable with LAE change patterns in the fully adjusted model.

To assess the possibility of reverse confounding between LAE and MCI reversion, people who can do more leisure activity may have less severe MCI (than others with similar CMMSE scores) and therefore, a better chance of reversion. We included the baseline MMSE score in the multivariate Cox hazard model for the sensitivity analysis.

Analyses were performed by using IBM SPSS v26.0 and R 4.2.1. Statistical tests were two-sided, and the *p*-values of < 0.05 were considered to indicate statistical significance.

## Results

### Baseline characteristics

Among 3,544 participants, the mean age was 86.7 years (SD, 9.6 years) at the baseline and 30.1% of participants were male. Over a mean of 3.8 years (SD, 1.7 years) of follow-up, 1,742 participants (49.2%) reverted to normal cognitive function over 6,045 person-years. Table 1 presents the baseline characteristics of the participants by the LAE change patterns. Most participants were in the low–low group (599, 16.9%), and the low–high group had the least participants (115, 3.2%). Participants in the high–high group were more likely to be younger elderly, female, and have lower ADL and IADL scores. Supplementary Table S1 presents the baseline characteristics of the participants by the MCI reverting status.

**Table 1:** Baseline characteristics of older adults according to LAE change patterns.

	**Low-low**	**Low-medium**	**Low-high**	**Medium-low**	**Medium-medium**	**Medium-high**	**High-low**	**High-medium**	**High-high**	***P*-value**
Number of participants	500	153	115	494	313	279	376	309	599	
Age in years	93.1 ± 7.4	88.7 ± 8.1	86.4 ± 9.0	91.05 ± 7.9	86.3 ± 8.4	83.7 ± 9.7	89.5 ± 8.7	84.3 ± 9.6	79.4 ± 8.5	<0.001***
Age group in years										<0.001***
Younger elderly	20 (4.0)	18 (11.8)	22 (19.1)	37 (7.5)	58 (18.5)	91 (32.6)	42 (11.2)	93 (30.1)	322 (53.8)	
Octogenarian	140 (28.0)	63 (41.2)	53 (46.1)	170 (34.4)	140 (44.7)	115 (41.2)	136 (36.2)	122 (39.5)	201 (33.6)	
Nonagenarian	340 (68.0)	72 (47.1)	40 (34.8)	287 (58.1)	115 (36.7)	73 (26.2)	198 (52.7)	94 (30.4)	76 (12.7)	
Sex										
Male	115 (23.0)	49 (32.0)	47 (40.9)	122 (24.7)	104 (33.2)	99 (35.5)	105 (27.9)	108 (35.0)	213 (35.6)	<0.001***
Educational level										
Years of schooling	1.3 ± 8.9	0.8 ± 2.2	2.8 ± 13.1	1.1 ± 6.2	1.0 ± 5.3	1.9 ± 10.0	1.3 ± 1.0	1.6 ± 6.3	1.4 ± 2.8	0.335
Location of residence										
Urban residence	181 (36.2)	56 (36.6)	30 (26.1)	208 (42.1)	107 (34.2)	92 (33.0)	134 (35.6)	120 (38.8)	215 (35.9)	0.054
Marital status										<0.001***
Married	66 (13.2)	39 (25.5)	37 (32.2)	69 (14.0)	70 (22.4)	83 (29.7)	82 (21.8)	93 (30.1)	267 (44.6)	
Divorced/widowed /never	407 (81.9)	114 (74.5)	78 (67.8)	425 (86.0)	242 (77.6)	196 (70.3)	294 (78.2)	216 (69.9)	332 (55.4)	
Economic status										0.017*
Favorable	407 (81.9)	117 (76.5)	86 (74.8)	404 (82.1)	232 (74.6)	205 (73.7)	308 (82.1)	237 (76.7)	470 (78.9)	
Unfavorable	90 (18.1)	36 (23.5)	29 (25.2)	88 (17.9)	79 (25.4)	73 (26.3)	67 (17.9)	72 (23.3)	126 (21.1)	
Number of people living with	2.8 ± 2.2	2.5 ± 2.2	2.7 ± 2.2	2.7 ± 2.3	2.4 ± 2.3	2.3 ± 2.0	2.9 ± 5.6	2.4 ± 2.2	2.3 ± 2.2	0.005**
Living pattern										<0.001***
Living with family members	420 (84.2)	117 (76.5)	92 (80.0)	388 (78.7)	214 (68.4)	215 (77.1)	307 (82.1)	242 (78.3)	476 (79.1)	
Alone	69 (13.8)	29 (19.0)	21 (18.3)	89 (18.1)	81 (25.9)	56 (20.1)	60 (16.0)	60 (19.4)	118 (19.7)	
At nursing home	10 (2.0)	7 (4.6)	2 (1.7)	16 (3.2)	18 (5.8)	8 (2.9)	7 (1.9)	7 (2.3)	7 (1.2)	
Functional status										
ADL	7.2 ± 2.3	6.7 ± 1.7	6.6 ± 1.4	6.5 ± 1.2	6.3 ± 0.8	6.2 ± 0.7	6.3 ± 1.0	6.2 ± 0.8	6.1 ± 0.4	<0.001***
IADL	17.9 ± 5.0	15.8 ± 5.3	15.2 ± 5.1	14.7 ± 4.6	12.7 ± 4.1	12.3 ± 4.2	12.5 ± 4.1	10.9 ± 3.3	10.1 ± 2.7	<0.001***
Lifestyle										
Smoke at present	53 (10.6)	24 (15.7)	16 (13.9)	67 (13.6)	52 (16.7)	38 (13.7)	59 (15.7)	56 (18.2)	155 (25.9)	<0.001***
Drink at present	66 (13.2)	17 (11.2)	14 (12.3)	60 (12.2)	53 (16.9)	45 (46.2)	82 (21.9)	59 (19.1)	149 (24.9)	<0.001***
Exercise at present	39 (7.8)	21 (13.7)	14 (12.3)	138 (28.0)	80 (25.6)	64 (23.0)	135 (36.0)	118 (38.2)	229 (38.2)	<0.001***
Chronic disease										
Hypertension	91 (18.9)	35 (23.5)	25 (22.7)	106 (22.6)	69 (23.0)	55 (20.8)	73 (0.4)	68 (23.6)	132 (23.3)	0.772
Diabetes	9 (1.9)	1 (0.7)	1 (0.9)	8 (1.7)	8 (2.6)	3 (1.1)	6 (1.7)	6 (2.1)	12 (2.1)	0.869
Heart disease	25 (5.2)	13 (8.8)	7 (6.4)	34 (7.2)	21 (6.9)	11 (4.2)	39 (11.0)	26 (8.9)	51 (9.0)	0.026*
Stroke or CVD	22 (4.6)	5 (3.4)	6 (5.3)	25 (5.4)	14 4.6)	10 (3.8)	18 (5.0)	12 (4.2)	28 (4.9)	0.984
Cataract	55 (11.4)	23 (15.8)	9 (8.0)	49 (10.4)	39 (13.0)	24 (9.1)	34 (9.6)	32 (11.0)	47 (8.3)	0.188
Digestive system diseases	21 (4.6)	6 (4.2)	8 (7.3)	23 (5.3)	10 (3.4)	10 (3.9)	17 (4.9)	17 (6.0)	31 (5.6)	0.768
Arthritis	86 (17.8)	23 (15.6)	22 (20.0)	96 (20.4)	64 (21.0)	55 (0.6)	75 (20.8)	69 (23.4)	122 (21.4)	0.627
Parkinson's disease	4 (0.9)	1 (0.7)	0 (0)	3 (0.7)	3 (1.0)	2 (0.8)	2 (0.6)	0 (0)	2 (0.4)	0.815

* *P* < 0.05

** *P* < 0.01

*** *P* < 0.001.

### Dose-response association between LAE change points and MCI reversion

**Figure 2:**
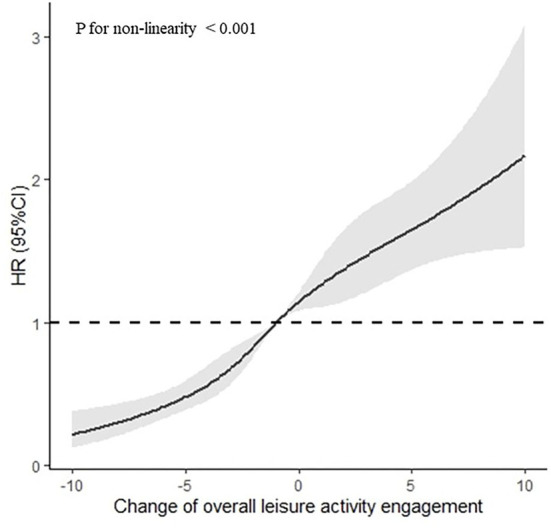
Association between the LAE change points and MCI reversion rate based on restricted cubic spline model. The results derived from the full-adjusted models were presented as hazard ratios with 95% confidential intervals. The model adjusted for sex, age, residence, years of schooling, marital status, economic status, living pattern, number of people living with, smoking, alcohol drinking, activity of daily living (ADL), instrumental activity of daily living (IADL), and chronic disease (such as hypertension, diabetes, heart disease, stroke or CVD, cataract, digestive system diseases, arthritis, and Parkinson's disease).

After adjusting for sex, age, residence, years of schooling, marital status, economic status, living pattern, number of people living with, smoking, alcohol drinking, ADL, IADL, and chronic disease (such as hypertension, diabetes, heart disease, stroke or CVD, cataract, digestive system diseases, arthritis, and Parkinson's disease), the restricted cubic spline model showed a nonlinear relationship between LAE change patterns and MCI reversion (Figure 2 nonlinear test, χ2 = 157.97, *P*_non−linearity_ <0.001).

### LAE change patterns and MCI reversion

**Figure 3:**
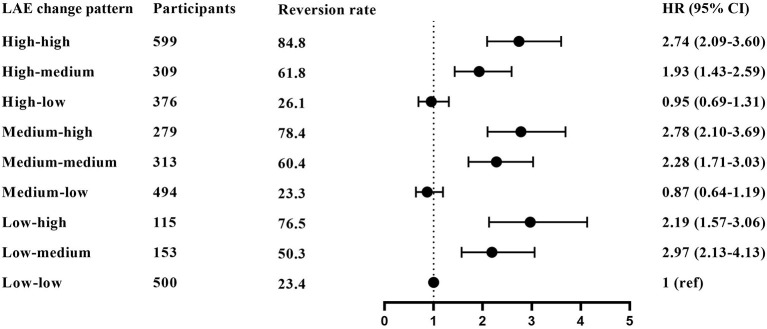
The association between overall LAE change patterns and MCI reversion. The results derived from the full-adjusted models were presented as hazard ratios with 95% confidential intervals. The model adjusted for sex, age, residence, years of schooling, marital status, economic status, living pattern, number of people living with, smoking, alcohol drinking, activity of daily living (ADL), instrumental activity of daily living (IADL), and chronic disease (such as hypertension, diabetes, heart disease, stroke or CVD, cataract, digestive system diseases, arthritis, and Parkinson's disease).

The high–high group has the highest MCI reversion rate at 84.8%, and the low–low group has the lowest reversion rate at 23.4%. Figure 3 and Supplementary Table S2 show the association between overall LAE change patterns and the reversion rate. Taking participants in the low–low group as a reference, participants in the low–medium, low–high, medium–medium, medium–high, high–medium, and high–high groups had increased possibilities of MCI reversion with HRs (95% CI) of 2.19 (1.57–3.06), 2.97 (2.13–4.13), 2.28 (1.71–3.03), 2.78 (2.10–3.69), 1.93 (1.43–2.59), and 2.74 (2.09–3.60), respectively.

The association among cognitively stimulating, physically active/demanding, and socially engaged LAE change patterns and MCI reversion is also shown in Table 2. The associations between cognitively stimulating LAE change patterns and MCI reversion were similar to the main results, as physically active/demanding LAE change patterns. However, physically active/demanding LAE showed a larger effect on MCI reversion than cognitively stimulating LAE. For social-based LAE change patterns, participants in the medium–medium group and the high–medium group did not have increased possibilities of MCI reversion with HRs (95% CI) of 1.26 (0.75–2.11) and 1.30 (0.94–1.80), respectively.

**Table 2:** The association between cognitively stimulating, physically active/demanding, and socially engaged LAE change patterns and MCI reversion.

	**Low-low**	**Low-medium**	**Low-high**	**Medium-low**	**Medium-medium**	**Medium-high**	**High-low**	**High-medium**	**High-high**
**Cognitively stimulating**									
No of reverters/ person years	195/2,604	258/1,575	27/109	225/3,250	799/4,533	83/351	12/210	47/250	72/267
Reversion rate	28.6	59.3	79.4	28.6	64.5	79.0	21.4	65.3	87.8
Model 1	1 ref	1.85 (1.53–2.24)***	2.60 (1.73–3.90)***	0.85 (0.70–1.03)	1.88 (1.60–2.21)***	2.32 (1.78–3.03)***	0.65 (0.36–1.16)	1.95 (1.41–2.70)***	2.51 (1.90–3.33)***
Model 2	1 ref	1.94 (1.57–2.39)***	2.74 (1.78–4.21)***	0.89 (0.72–1.10)	1.98 (1.65–2.38)***	2.45 (1.82–3.29)***	0.74 (0.40–1.37)	2.04 (1.44–2.89)***	2.65 (1.95–3.61)***
Model 3	1 ref	1.88 (1.50–2.35)***	2.57 (1.61–4.11)***	0.86 (0.68–1.09)	1.90 (1.56–2.32)***	2.22 (1.60–3.08)***	0.81 (0.44–1.51)	1.78 (1.20–2.65)**	2.53 (1.80–3.57)***
**Physically active/demanding**									
No of reverters/ person years	69/1,147	66/511	51/212	137/2,083	289/1,759	290/1,189	74/1,278	243/1,497	385/1,666
Reversion rate	21.8	42.9	77.3	25.0	57.0	84.5	23.7	58.8	79.7
Model 1	1 ref	1.93 (1.38–2.71)***	3.18 (2.21–4.59)***	1.02 (0.76–1.36)	2.24 (1.72–2.92)***	2.96 (2.25–3.88)***	0.87 (0.63–1.21)	2.13 (1.62–2.80)***	2.79 (2.14–3.65)***
Model 2	1 ref	1.91 (1.34–2.74)***	3.24 (2.19–4.79)***	1.04 (0.76–1.41)	2.27 (1.71–3.00)***	2.93 (2.20–3.91)***	0.90 (0.63–1.28)	2.08 (1.55–2.78)***	2.84 (2.14–3.77)***
Model 3	1 ref	1.86 (1.27–2.73)**	3.20 (2.10–4.88)***	0.97 (0.69–1.35)	2.13 (1.56–2.91)***	2.61 (1.89–3.60)***	0.85 (0.57–1.25)	1.93 (1.39–2.69)***	2.62 (1.90–3.63)***
**Socially engaged**									
No of reverters/ person years	589/6,016	91/460	211/1,001	82/895	20/112	57/261	246/2,469	65/370	355/1,546
Reversion rate	36.6	72.2	72.8	35.8	62.5	76.0	41.7	71.4	80.5
Model 1	1 ref	1.78 (1.43–2.22)***	1.85 (1.58–2.17)***	0.90 (0.71–1.13)	1.36 (0.87–2.13)	1.75 (1.33–2.30)***	0.94 (0.80–1.09)	1.42 (1.09–1.84)**	1.70 (1.47–1.96)***
Model 2	1 ref	1.94 (1.53–2.48)***	1.95 (1.64–2.32)***	0.93 (0.73–1.20)	1.28 (0.77–2.11)	1.82 (1.35–2.46)***	0.97 (0.83–1.15)	1.41 (1.07–1.86)*	1.79 (1.54–2.09)***
Model 3	1 ref	2.05 (1.58–2.65)***	1.98 (1.64–2.40)***	0.96 (0.73–1.26)	1.26 (0.75–2.11)	1.77 (1.29–2.43)***	0.95 (0.79–1.14)	1.30 (0.94–1.80)	1.67 (1.40–1.98)***

* *P* < 0.05

** *P* < 0.01

*** *P* < 0.001.

Model 1 Adjusted for age (continuous), gender.

Model 2 Adjusted for model 1 plus residence, years of schooling, marital status, economic status, living pattern, number of people living with.

Model 3 Adjusted for model 2 plus tobacco smoking, alcohol drinking, regular exercise, ADL, IADL, chronic disease (hypertension, diabetes, heart disease, stroke or CVD, cataract, digestive system disease, arthritis, Parkinson's disease).

In addition, the associations between absolute LAE change patterns and MCI reversion in eight activities are shown in Table 3. For most of the leisure activities, compared with participants in the rarely–rarely group, participants in the rarely–frequently, and frequently–frequently groups had increased possibilities of MCI reversion.

**Table 3:** The association between absolute LAE change patterns and MCI reversion in eight activities.

	**Rarely-rarely**	**Rarely-occasionally**	**Rarely-frequently**	**Occasionally -rarely**	**Occasionally-occasionally**	**Occasionally -frequently**	**Frequently-rarely**	**Frequently-occasionally**	**Frequently-frequently**
Cognitive activity									
*Read newspapers/books*									
No of reverters/ person years	1,436/11,693	46/212	74/322	37/258	8/24	15/61	41/340	12/46	49/193
Reversion rate	46.9	71.9	77.1	51.4	100.0	83.3	44.1	80.0	83.1
HR (95%CI)	1 ref	1.53 (1.10–2.13) *	1.49 (1.12–1.98) **	1.04 (0.71–1.52)	1.84 (0.87–3.91)	1.74 (0.89–3.43)	0.88 (0.61–1.26)	1.69 (0.87–3.29)	1.62 (1.16–2.27) **
*Watch TV and/or listen to radio*									
No of reverters/ person years	2,078/2,706	53/358	232/1,335	60/817	39/203	138/730	180/2,615	83/630	728/3,767
Reversion rate	29.1	54.1	62.4	30.2	65.0	71.5	28.3	49.7	69.0
HR (95%CI)	1 ref	1.74 (1.22–2.46) **	1.93 (1.54–2.42) ***	0.93 (0.66–1.31)	2.31 (1.57–3.41) ***	1.87 (1.44–2.43) ***	0.81 (0.64–1.03)	1.59 (1.18–2.16) **	1.93 (1.58–2.35) ***
Physical activity									
*Housework*									
No of reverters/ person years	308/3,840	31/226	164/752	59/735	9/54	54/205	258/3,329	42/333	796/3,689
Reversion rate	29.8	46.3	73.5	30.4	52.9	87.1	32.7	50.6	77.7
HR (95%CI)	1 ref	1.67 (1.12–2.50) *	2.31 (1.85–2.89) ***	0.96 (0.70–1.32)	2.04 (0.96–4.35)	2.79 (2.03–3.84) ***	0.92 (0.74–1.13)	1.54 (1.05–2.26) *	2.23 (1.84–2.70) ***
*Personal outdoor activities*									
No of reverters/ person years	185/2,181	43/268	265/1,264	61/707	31/149	106/503	255/2,813	91/563	567/2,877
Reversion rate	30.8	53.8	72.4	32.2	72.1	74.6	34.9	58.7	68.1
HR (95%CI)	1 ref	1.65 (1.12–2.43) *	1.86 (1.49–2.32) ***	1.02 (0.73–1.41)	2.02 (1.34–3.05) **	1.73 (1.30–2.31) ***	0.92 (0.73–1.16)	1.42 (1.06–1.91) *	1.76 (1.43–2.16) ***
*Garden work*									
No of reverters/ person years	1,285/10,771	46/216	152/647	66/456	3/11	8/24	94/750	14/57	50/221
Reversion rate	26.2	30.6	29.5	26.5	36.4	33.3	26.3	29.8	29.4
HR (95%CI)	1 ref	1.45 (1.04–2.01) *	1.39 (1.31–1.70) **	1.10 (0.83–1.47)	1.80 (0.25–12.85)	2.21 (0.98–4.96)	0.87 (0.68–1.10)	1.54 (0.87–2.73)	1.38 (0.98–1.94)
Social activity									
*Raise domestic animals*									
No of reverters/ person years	908/8,080	47/240	161/750	76/609	3/25	33/145	214/2,090	30/161	248/1,057
Reversion rate	42.0	71.2	71.9	48.7	37.5	78.6	44.0	71.4	81.3
HR (95%CI)	1 ref	1.66 (1.20–2.29) **	1.72 (1.42–2.09) ***	1.12 (0.86–1.48)	1.04 (0.33–3.23)	1.69 (1.13–2.54) *	0.84 (0.71–1.01)	1.18 (0.76–1.84)	1.46 (1.22–1.74) ***
*Play cards and/or mahjong*									
No of reverters/ person years	1,347/11,062	58/262	68/314	62/446	10/55	22/92	68/523	16/92	66/300
Reversion rate	46.1	74.4	80.1	48.8	66.7	81.5	51.9	66.7	81.5
HR (95%CI)	1 ref	1.47 (1.09–1.99) *	1.64 (1.21–2.23) **	1.02 (0.75–1.38)	1.20 (0.59–2.42)	1.26 (0.79–1.99)	1.03 (0.79–1.35)	0.91 (0.51–1.62)	1.48 (1.10–1.98) **
*Social activities (organized)*									
No of reverters/ person years	1,353/11,123	144/667	58/254	82/606	17/71	9/52	35/293	14/53	5/16
Reversion rate	46.1	77.4	85.3	49.7	89.5	75.0	43.4	93.3	100.0
HR (95%CI)	1 ref	1.41 (1.15–1.73) **	2.07 (1.50–2.84) ***	1.03 (0.79–1.34)	1.46 (0.82–2.59)	1.30 (0.61–2.76)	0.89 (0.60–1.32)	1.62 (0.83–3.15)	1.70 (0.70–4.14)

* *P* < 0.05

** *P* < 0.01

*** *P* < 0.001.

Adjusted for age (continuous), gender, residence, years of schooling, marital status, economic status, living pattern, number of people living with, tobacco smoking, alcohol drinking, regular exercise, ADL, IADL, chronic disease (hypertension, diabetes, heart disease, stroke or CVD, cataract, digestive system disease, arthritis, Parkinson's disease).

### Stratified analysis

In the stratified analysis (Table 4), among participants with unfavorable economic status, compared with those in the low–low group, only participants in the medium–high and high–high groups had increased possibilities with HRs (95% CI) of 2.44 (1.29–4.60) and 2.17 (1.18–3.98), respectively. Among participants living alone or living at nursing homes, the association between LAE change patterns and MCI reversion was insignificant. Among participants who were divorced/widowed/never married, compared with participants in the low–low group, participants in the low–medium, low–high, medium–medium, medium–high, and high–high groups had increased possibilities with HRs (95%CI) of 2.45 (1.65–3.62), 3.30 (2.20–4.93), 2.19 (1.56–3.07), 2.86 (2.04–4.00), 1.88 (1.32–2.70), and 3.01 (2.17–4.17), respectively. In addition, LAE presented a larger effect on MCI reversion among urban residents than rural residents.

**Table 4:** The association between LAE change patterns and MCI reversion in subgroups.

	**Low-low**	**Low-medium**	**Low-high**	**Medium-low**	**Medium-medium**	**Medium-high**	**High-low**	**High-medium**	**High-high**
Age									
*Younger elderly*									
No of reverters/ person years	6/72.0	11/56	19/78	11/150	43/179	76/302	15/147	61/341	274/1,030
Reversion rate	30.0	64.7	86.4	30.6	81.1	90.5	40.5	73.6	90.4
HR (95%CI)	1 ref	1.62 (0.53–5.01)	1.70 (0.57–5.07)	0.50 (0.15–1.67)	1.88 (0.67–5.24)	1.92 (0.70–5.28)	1.05 (0.35–3.10)	1.41 (0.51–3.86)	1.94 (0.72–5.20)
*Octogenarian*									
No of reverters/ person years	43/505	33/190	42/168	42/639	85/469	86/375	38/616	66/448	150/640
Reversion rate	32.1	57.9	80.8	25.8	64.9	78.9	28.6	57.9	80.6
HR (95%CI)	1 ref	2.23 (1.36–3.65) **	2.86 (1.76–4.65) ***	0.77 (0.48–1.26)	1.98 (1.29–3.05) **	2.42 (1.58–3.70) ***	0.65 (0.39–1.09)	1.52 (0.95–2.42)	2.37 (1.56–3.60) ***
*Nonagenarian*									
No of reverters/ person years	50/1,181	20/230	26/140	48/997	42/383	38/211	34/705	43/305	46/245
Reversion rate	15.2	30.3	65.0	17.1	38.2	56.7	17.4	47.8	63.9
HR (95%CI)	1 ref	2.14 (1.23–3.70) **	3.48 (1.96–6.20) ***	1.02 (0.66–1.59)	2.22 (1.38–3.55) **	3.22 (1.94–5.36) ***	1.15 (0.71–1.87)	2.31 (1.36–3.93) **	3.67 (2.24–6.03) ***
Sex									
*Male*									
No of reverters/ person years	34/386	20/152	36/143	28/420	61/357	78/302	26/386	71/351	178/665
Reversion rate	30.4	44.4	78.3	23.1	61.6	84.8	25.2	69.6	87.7
HR (95%CI)	1 ref	1.41 (0.79–2.52)	2.37 (1.40–4.04) **	0.62 (0.35–1.09)	1.62 (1.01–2.61) *	2.29 (1.45–3.63) ***	0.71 (0.41–1.24)	1.67 (1.02–2.71) *	2.10 (1.34–3.27) **
*Female*									
No of reverters/ person years	65/1,371	44/324	51/242	73/1,366	109/674	122/587	61/1,082	99/743	292/1,250
Reversion rate	17.6	46.3	75.0	20.3	55.9	72.6	23.3	53.2	81.6
HR (95%CI)	1 ref	2.75 (1.82–4.15) ***	3.47 (2.26–5.31) ***	1.01 (0.69–1.48)	2.68 (1.87–3.84)	2.99 (2.08–4.29) ***	1.08 (0.73–1.61)	2.02 (1.39–2.94) ***	3.10 (2.20–4.37) ***
Economic status									
*Favorable*									
No of reverters/ person years	76/1,446	52/366	69/286	84/1,437	130/754	144/653	71/1,205	129/827	366/1,456
Reversion rate	19.2	48.1	81.2	21.5	60.2	74.6	23.9	58.6	84.3
HR (95%CI)	1 ref	2.38 (1.63–3.48) ***	3.18 (2.19–4.61) ***	0.93 (0.66–1.32)	2.41 (1.75–3.33) ***	2.85 (2.08–3.93) ***	0.96 (0.67–1.38)	1.97 (4.41–2.74) ***	2.86 (2.11–3.89) ***
*Unfavorable*									
No of reverters/ person years	84/303	32/110	29/100	87/344	76/261	66/233	67/260	68/267	124/447
Reversion rate	25.0	37.5	62.1	18.4	51.3	83.3	23.9	60.3	81.5
HR (95%CI)	1 ref	1.44 (0.68–3.05)	2.00 (0.94–4.25)	0.61 (0.30–1.25)	1.71 (0.91–3.22)	2.44 (1.29–4.60) **	0.89 (0.42–1.90)	1.83 (0.93–3.58)	2.17 (1.18–3.98) *
Marital status									
*Married*									
No of reverters/ person years	17/222	18/122	29/114	16/253	47/211	69/268	31/316	59/304	225/844
Reversion rate	27.9	50.0	78.4	23.9	75.8	89.6	39.2	71.2	88.6
HR (95%CI)	1 ref	1.54 (0.80–2.98)	2.26 (1.23–4.16) **	0.57 (0.28–1.14)	2.19 (1.25–3.83) **	2.32 (1.34–4.02) **	1.05 (0.58–1.91)	1.75 (1.00–3.07)	2.19 (1.30–3.70) **
*Divorced/ widowed /never*									
No of reverters/ person years	82/1,536	46/354	58/272	85/1,533	123/817	131/621	56/1,152	111/790	245/1,071
Reversion rate	19.5	44.2	75.3	20.6	53.2	71.6	19.6	54.1	79.8
HR (95%CI)	1 ref	2.45 (1.65–3.62) ***	3.30 (2.20–4.93) ***	0.96 (0.68–1.36)	2.19 (1.56–3.07) ***	2.86 (2.04–4.00) ***	0.84 (0.57–1.24)	1.88 (1.32–2.70) **	3.01 (2.17–4.17) ***
Living pattern									
*Living with family members*									
No of reverters/ person years	77/1,471	51/360	69/310	76/1,385	117/687	152/682	71/1,185	129/847	379/1,510
Reversion rate	18.9	47.2	75.8	20.0	59.3	75.2	23.7	57.6	85.2
HR (95%CI)	1 ref	2.36 (1.64–3.40) ***	3.23 (2.28–4.59) ***	0.88 (0.62–1.23)	2.60 (1.91–3.54) ***	3.05 (2.25–4.14) ***	1.02 (0.72–1.45)	2.12 (1.54–2.92) ***	3.06 (2.28–4.10) ***
*Alone or at nursing home*									
No of reverters/ person years	21/28.8	13/40.6	18/78.3	25/25.3	53/54.6	48/82.8	16/25.0	41/64.1	90/78.3
Reversion rate	284	116	76	399	344	207	276	247	402
HR (95%CI)	1 ref	1.28 (0.53–3.11)	1.46 (0.45–4.70)	0.80 (3.56–1.80)	1.11 (0.49–2.50)	1.37 (0.62–3.04)	0.61 (0.25–1.51)	1.06 (0.46–2.44)	1.24 (0.57–2.68)

* *P* < 0.05

** *P* < 0.01

*** *P* < 0.001.

Adjusted for age (continuous), gender, residence, years of schooling, marital status, economic status, living pattern, number of people living with, tobacco smoking, alcohol drinking, regular exercise, ADL, IADL, chronic disease (hypertension, diabetes, heart disease, stroke or CVD, cataract, digestive system disease, arthritis, Parkinson's disease).

When stratified by age (Supplementary Table S3), the associations between overall LAE and MCI reversion were insignificant among the younger elderly. For cognitively stimulating LAE, compared to younger elderly in the low–low group, those in the low–high and high–high groups had increased possibilities of MCI reversion with HRs (95% CI) of 2.61 (1.23–5.55) and 1.96 (1.09–3.52), respectively. In addition, an octogenarian in the medium–low group had a statistically significant lower possibility of MCI reversion with an HR (95% CI) of 0.59 (0.41–0.85). For physically active/demanding LAE, compared with the low–low group, nonagenarians in the medium–medium, medium–high, and high–high groups had increased possibilities of MCI reversion with HRs (95% CI) of 1.71 (1.09–2.70), 2.98 (1.82–4.90), 2.72 (1.55–4.78), respectively. The associations between overall physically active/demanding and MCI reversion were not significant among the younger elderly. For socially engaged LAE, only nonagenarians did not show a statistically significant result in the medium–medium group.

When stratified by sex (Supplementary Table S4), the associations between different types of LAE change patterns and MCI reversion among female participants were similar to the main results. For physically active/demanding LAE, male participants in the low–high, medium–high, and high–high groups have higher possibilities of MCI reversion with HRs (95% CI) of 2.32 (1.22–4.40), 1.95 (1.17–3.24), and 3.28 (1.73–6.23), respectively.

### Sensitivity analysis

Supplementary Table S5 shows the associations among overall, cognitively stimulating, physically active/demanding, and socially engaged LAE changes patterns and MCI reversion after setting MMSE change≥4 points as MCI reverted status. All of the associations were similar to results when setting MMSE change≥3 points as MCI reverted to normal cognitive function status.

Supplementary Table S6 shows the associations between all variables and MCI reversion after adding the baseline MMSE score as a covariate. All of the associations were similar to the main results. Taking participants in the low–low group as a reference, participants in the low–medium, low–high, medium–medium, medium–high, high–medium, and high–high groups had increased possibilities of MCI reversion with HRs (95% CI) of 2.42 (1.64–3.58), 3.58 (2.49–5.15), 2.80 (2.02–3.89), 3.34 (2.42–4.62), 2.36 (1.68–3.31), and 3.46 (2.54–4.73), respectively. Meanwhile, the baseline MMSE score was not significantly associated with MCI reversion.

## Discussion

In this prospective study, we found that participants who maintained the highest level of LAE had the greatest possibility of MCI reversion. A higher level of LAE maintenance was also associated with the increased possibility of MCI reversion. In contrast, maintaining lower LAE was not associated with MCI reversion among older adults in subsequent years. The results of this study could provide practical instructions to older adults on how dynamic changes in LAE are associated with cognitive ability. These findings highlighted the significance of addressing higher LAE among older adults for promoting MCI reversion in later life.

Taking the general association between LAE and cognitive ability first, the current findings replicated those widely reported in the literature. Individuals participating in more leisure activities generally score higher on cognitive ability tests. However, the association between LAE changes over time and MCI reversion is still unclear. Our study found that participants with MCI who maintained higher LAE had a greater possibility to reverse to normal cognitive function. These findings were in line with the previous study, which suggested that community-dwelling older adults with MCI who continued their multi-domain lifestyle activities (such as cognitive, social, and productive) were more likely to revert to normal cognitive function (31). However, this previous study did not investigate which level of LAE is mostly beneficial for cognitive ability. Our study showed that maintaining the highest level of most types of LAE is positively associated with MCI reversion in various populations, which has important public health implications for older adults.

Results from the subgroup analysis of three sub-types of LAE showed that physically active/ demanding LAE had the most robust association with a higher possibility of MCI reversion compared with cognitively stimulating and socially engaged LAE. Previous studies have shown that physical activities were positively associated with a reduced risk of cognitive decline and dementia (32). For example, in a Swedish cohort, physical activity positively predicted changes in cognitive ability (33). In older adults of the offspring cohort, total physical activity (measured in steps/day) was associated with better executive function (34). In addition, the positive effect of high-intensity exercise on cognitive ability was also reported in some interventional studies (35, 36). Some evidence showed that household physical activity benefits the brain volume, especially the gray matter volume, which might delay the decline in memory function and executive control process among older adults (37). Physical activity, which might more directly contribute to the levels of brain structure and function than cognitive and social activities among older adults with MCI (38, 39), may be able to stop the neuronal decline caused by age and aid in the growth of capillaries in the brain (40).

Meanwhile, our findings also reinforced the arguments that changing to or maintaining a higher level of cognitive-based LAE is beneficial for MCI reversion. Even though previous findings suggested that more frequent cognitive leisure activities were associated with a higher level of cognitive function in later life (41, 42), few reported the association between changes in cognitively stimulating LAE and cognitive ability. Our study found that if a participant changed their frequency of watching TV and/or listening to the radio from “low–low” to “low–high,” the possibilities of MCI reversion of this participant will increase. This goal is achievable for almost all older people since these activities are frequently carried out daily. Intellectual stimulation has been found to strengthen synaptic transmission (i.e., neural plasticity) and increase cognitive reserve (43). Therefore, it might be desirable for older adults with MCI to participate in more cognitive leisure activities later in life.

Another interesting finding was that even though the MCI reversion rates of younger elderly were higher than octogenarians and nonagenarians, the impact of LAE on MCI reversion was more evident among octogenarians and nonagenarians than younger elderly. We have also found that the impact of improvement in cognitively stimulating LAE on MCI reversion was most evident in nonagenarians, who were nonagenarian adults. Some studies have reported that younger age was positively associated with the possibility of MCI reversion (5, 26). However, few studies investigated the moderating effect of age on the relationship between leisure activity and MCI reversion. Bielak et al. found that nonagenarian adults showed a stronger relationship between cognitive-based LAE level and cognitive ability than young–old adults (44). This finding may indicate that there appears to be something unique about how cognitive-based LAE and perceptual speed ability change over time for nonagenarian individuals compared with young–old individuals. Later-life LAE among older adults with MCI may predict later cognitive status, but individuals might respond differently to such effects (45). In addition, older adults with younger age tend to participate in more challenging activities than those in later older years. In fact, the nonagenarian adults are more likely to preoccupy themselves with passive activities, such as watching TV or listening to the radio (46). The results implicated that an age-stratified policy to prevent the decline of cognitive function while promoting lifestyle adjustment is needed in older Chinese adults. For example, simple cognitive leisure activities are encouraged in nonagenarian adults to improve their cognitive functions. However, Ihle et al. suggested that mental leisure activity only supported cognitive ability in young–old individuals (47). Another possible reason for the different relationships between LAE and cognitive ability among older adults in different age groups was that nonagenarians accounted for the largest proportion of participants, which might pose significant results. Age is typically overlooked as a possible moderator in the relationship between LAE and cognitive ability. More studies are necessary to determine how the relationship between LAE and cognitive ability differ according to age.

The leisure activity engagement-cognitive ability relations may also be modified by sex. In our study, the relations were less significant in male participants than in female participants. Compared to maintaining low–low physically active/demanding LAE, only maintaining or changing to a high level of participation could increase the possibility of MCI reversion for male participants. The most recent work considered gender difference was on physical activity. Since activities included in physical-based LAE were mainly about domestic activity, which was usually finished by older female adults under traditional Chinese culture (48). As noted by Biek (49), gender differences in LAE-cognitive ability relations may be partially explained by traditional roles (50). Fellendorf et al. also found that female individuals in the vigorous physical activity group performed significantly higher in most cognitive domains than female individuals with moderate or low physical activity (51). However, this significant difference was not found in male participants. It is important to consider gender-specific intervention in leisure activities to improve cognitive function among older adults.

We also found that compared to older adults with unfavorable economic status, those with favorable economic status were more likely to revert to normal cognitive function when they increased their LAE. In addition, older adults who lived with family members had a higher possibility of MCI reversion than older adults who lived alone or stayed at nursing homes. Meanwhile, LAE presented a larger effect on MCI reversion among urban residents than rural residents. These results showed that older adults of higher socioeconomic status could enjoy extra benefits from leisure activities. A recent study detected a substantial interaction between life course economic status and LAE on cognitive function (52). Even though older adults selected the same activity category, those with higher socioeconomic status might choose specific types that were more cognitively engaging. For example, Di Liegro et al. (53) reported that the brain-derived neurotrophic factor (BDNF), which is positively related to cognition, tends to be elevated more by open-skill exercise (e.g., basketball and badminton) than by closed-skill exercise (e.g., walking and running), probably due to additional attention required when facing ever-changing situations (53). However, open-skill exercise requires specific equipment and facilities, which needs investment. Therefore, older adults with higher socioeconomic status may benefit more from LAE.

Even though this study provides clear implications for the reversion from MCI to normal cognitive function in Chinese older adults, some limitations still exist. In this study, MCI was measured by MMSE and its sensitivity in screening MCI is lower than Montreal Cognitive Assessment (MOCA). However, MMSE still has comparable performance in the detection of MCI with 0.62 sensitivity and 0.87 specificity in the Chinese population (54). As the definition of MCI in this study was a bit different from the diagnostic criteria by Petersen et al. (55), participants without IADL disability at the baseline were used to assess sensitivity. The results were largely similar (Supplementary Table S7). The measure of leisure activities is also limited in its scope. Many activities not included, such as traveling, entertaining, and volunteering (56), and there is no measure of the intensity, duration, or quality of included activities. Meanwhile, the classification may not be perfectly mutually exclusive for some activities that can encompass one or more cognitively stimulating, physically active/demanding, and socially engaged domains. For example, playing Mahjong may fall into overlapping classes of cognitively stimulating and socially engaging activities, whereas raising domestic animals involves physical and social elements. However, the index of these leisure activities in each activity dimension could not be calculated without measuring the intensity, duration, and quality. Further studies are needed to investigate LAE in a more detailed way to avoid the underestimation or overestimation of effects (57).

In conclusion, maintaining or improving to a higher overall, cognitively stimulating, physically active/demanding, and socially engaged LAE was associated with a significantly higher MCI reversion rate in subsequent years among older adults. These results provided a practical message to the older adults about how dynamic changes in LAE were associated with improved cognitive function. Since the sample included in this study was mainly extremely aged, more studies between young and older adults were suggested. These findings also emphasized the importance of promoting a higher LAE across old age for preventing dementia in later life.

## Data availability statement

Publicly available datasets from The Chinese Longitudinal Healthy Longevity Survey (CLHLS)-Longitudinal Data repository were used in this study. These are available via, https://opendata.pku.edu.cn/dataset.xhtml?persistentId=doi:10.18170/DVN/WBO7LK. Further queries can be directed to the corresponding author.

## Ethics statement

The studies involving human participants were reviewed and the CLHLS study was approved by the Institutional Review Board of Duke University (Pro00062871) and the Biomedical Ethics Committee of Peking University (IRB00001052-13074). The patients/participants provided their written informed consent to participate in this study.

## Author contributions

QT contributed to the study design, interpretation of the findings, and revision of the manuscript. XX contributed to data analysis, interpretation of the findings, and drafting of the manuscript. SW contributed interpretation of the findings and revision of the manuscript. LN contributed to data cleaning and revision of the manuscript. IL contributed to data analysis and revision of the manuscript. All authors contributed to the article and approved the submitted version.

## Funding

This study was funded by the National Natural Science Foundation of China (Grant nos. 72204075 and 72204228), Hebei Provincial Postdoctoral Science Foundation (Grant no. B2022003032), Postdoctoral Research Funding of Hebei Medical University, and Hebei Province Social Science Development Research Project (Grant no. 20220202303).

## Conflict of interest

The authors declare that the research was conducted in the absence of any commercial or financial relationships that could be construed as a potential conflict of interest.

## Publisher's note

All claims expressed in this article are solely those of the authors and do not necessarily represent those of their affiliated organizations, or those of the publisher, the editors and the reviewers. Any product that may be evaluated in this article, or claim that may be made by its manufacturer, is not guaranteed or endorsed by the publisher.
