# Supplementary material for: Association of leisure activity changes and reversion from mild cognitive impairment to normal cognitive function among older adults: A prospective cohort study
Source: Front Public Health. 2022 Nov 22;10:1035762. doi: 10.3389/fpubh.2022.1035762 (PMC9724021; doi:10.3389/fpubh.2022.1035762)
Supplement: Supplementary file 1 [file Data_Sheet_1.docx]

Supplementary Material

Supplementary Table S1. Baseline characteristics of older people according to reverting status

|  | Total | Not reverted | Reverted | P value |
| --- | --- | --- | --- | --- |
| Number of participants | 3544 | 1802 | 1742 |  |
| Age in years | 86.7±9.6 | 90.3±8.5 | 82.9±9.2 | <0.001*** |
| **Age group in years** |  |  |  | <0.001*** |
| Younger elderly | 819 (23.1) | 205 (11.4) | 614 (35.2) |  |
| Octogenarian | 1315 (37.1) | 606 (33.6) | 709 (40.7) |  |
| Nonagenarian | 1410 (39.8) | 991 (55.0) | 419 (24.1) |  |
| Male | 1066 (30.1) | 453 (25.1) | 613 (35.2) | <0.001*** |
| Years of schooling | 1.4±6.7 | 1.2±6.9 | 1.6±6.6 | 0.061 |
| Urban residence | 287 (8.2) | 154 (8.7) | 133 (7.8) | 0.325 |
| **Marital status** |  |  |  | <0.001*** |
| Married | 953 (26.9) | 329 (18.3) | 624 (35.8) |  |
| Divorced/ widowed /never | 2588 (73.1) | 1471 (81.7) | 1117 (64.2) |  |
| **Economic status** |  |  |  | 0.431 |
| Favorable | 2797 (79.2) | 1433 (79.7) | 1364 (78.7) |  |
| Unfavorable | 734 20.8 | 364 (20.3) | 370 (21.3) |  |
| Number of people living with | 2.5±2.8 | 2.7±3.3 | 2.3±2.1 | <0.001*** |
| **Living pattern** |  |  |  | 0.008** |
| Living with family members | 2755 (77.9) | 1427 (79.4) | 1328 (76.4) |  |
| Alone | 694 (19.6) | 319 (17.8) | 375 (21.6) |  |
| At nursing home | 87 (2.5) | 51 (2.80) | 36 (2.10 |  |
| ADL | 6.4±1.3 | 6.6±1.50 | 6.3±1.0 | <0.001*** |
| IADL | 13.3±4.9 | 14.5±5.1 | 12.0± 4.4 | <0.001*** |
| Smoke at present | 567 (16.0) | 234 (13.0) | 333 (19.2) | <0.001*** |
| Drink at present | 595 (16.8) | 278 (15.5) | 317 (18.2) | 0.031* |
| Exercise at present | 949 (26.8) | 469 (26.1) | 480 (27.6) | 0.324 |
| **Chronic disease** |  |  |  |  |
| Hypertension | 800 (23.7) | 380 (22.3) | 420 (25.2) | 0.052 |
| Diabetes | 75 (2.2) | 30 (1.8) | 45 (2.7) | 0.079 |
| Heart disease | 265 (7.9) | 127 (7.4) | 138 (8.3) | 0.371 |
| Stroke or CVD | 170 (5.0) | 94 (12.1) | 76 (4.6) | 0.209 |
| Cataract | 371 (11.0) | 206 (12.1) | 165 (9.9) | 0.048* |
| Digestive system diseases | 162 (5.0) | 72 (4.40 | 90 (5.6) | 0.125 |
| Arthritis | 690 (20.3) | 358 (20.9) | 332 (19.8) | 0.468 |
| Parkinson’s disease | 20 (0.6) | 10 (0.6) | 10 (0.6) | 0.935 |

*p<0.05 **p<0.01 ***p<0.001

Supplementary Table S2. The association between overall LAE change patterns and MCI reversion

|  | Low-low | Low-medium | Low-high | Medium-low | Medium-medium | Medium-high | High-low | High-medium | High-high |
| --- | --- | --- | --- | --- | --- | --- | --- | --- | --- |
| No of reverters/ person years | 117/1823 | 77/514 | 88/389 | 115/1834 | 189/1101 | 219/947 | 98/1507 | 191/1172 | 508/2041 |
| Reversion rate | 23.4 | 50.3 | 76.5 | 23.3 | 60.4 | 78.4 | 26.1 | 61.8 | 84.8 |
| Model 1 | 1 ref | 2.09 (1.57-2.79) *** | 3.00 (2.26-3.97) *** | 0.94 (0.73-1.22) | 2.30 (1.82-2.90) *** | 2.86 (2.26-3.61) *** | 0.92 (0.70-1.21) | 2.06 (1.62-2.61) *** | 2.85 (2.29-3.53) *** |
| Model 2 | 1 ref | 2.24 (1.64-3.05) *** | 3.02 (2.23-4.10) *** | 0.92 (0.70-1.23) | 2.31 (1.79-2.99) *** | 2.93 (2.28-3.77) *** | 0.94 (0.71-1.25) | 2.04 (1.58-2.63) *** | 2.91 (2.30-3.67) *** |
| Model 3 | 1 ref | 2.19 (1.57-3.06) *** | 2.97 (2.13-4.13) *** | 0.87 (0.64-1.19) | 2.28 (1.71-3.03) *** | 2.78 (2.10-3.69) *** | 0.95 (0.69-1.31) | 1.93 (1.43-2.59) *** | 2.74 (2.09-3.60) *** |

*p<0.05 **p<0.01 ***p<0.001

Model 1 Adjusted for age (continuous), gender

Model 2 Adjusted for model 1 plus residence, years of schooling, marital status, economic status, living pattern, number of people living with

Model 3 Adjusted for model 2 tobacco smoking, alcohol drinking, regular exercise, ADL, IADL, chronic disease (hypertension, diabetes, heart disease, stroke or CVD, cataract, digestive system disease, arthritis, Parkinson’s disease)

Supplementary Table S3. The association between cognitively stimulating, physically active/demanding, and socially engaged LAE change patterns and MCI reversion in age subgroups.

|  | Low-low | Low-medium | Low-high | Medium-low | Medium-medium | Medium-high | High-low | High-medium | High-high |
| --- | --- | --- | --- | --- | --- | --- | --- | --- | --- |
| **Cognitively stimulating** |  |  |  |  |  |  |  |  |  |
| Younger elderly | 1 ref | 1.44 (0.89-2.31) | 2.61 (1.23-5.55) * | 0.83 (0.50-1.37) | 1.51 (0.98-2.32) | 1.80 (0.97-3.33) | 0.71 (0.24-2.07) | 1.41 (0.72-2.78) | 1.96 (1.09-3.52) * |
| Octogenarian | 1 ref | 1.55 (1.11-2.16) * | 2.14 (0.91-5.04) | 0.59 (0.41-0.85) ** | 1.64 (1.23-2.18) ** | 1.75 (1.06-2.89) * | 0.43 (0.13-1.40) | 1.31 (0.68-2.55) | 2.52 (1.47-4.30) ** |
| Nonagenarian | 1 ref | 2.98 (1.96-4.54) *** | 3.73 (1.43-9.75) ** | 1.39 (0.92-2.11) | 2.75 (1.89-4.02) *** | 3.50 (1.79-6.86) *** | 1.41 (0.49-4.03) | 3.44 (1.53-7.76) ** | 3.19 (1.30-7.85) * |
| **Physically active/demanding** |  |  |  |  |  |  |  |  |  |
| Younger elderly | 1 ref | 1.28 (0.37-4.44) | 1.68 (0.50-5.60) | 0.42 (0.12-1.43) | 1.27 (0.45-3.59) | 1.27 (0.45-3.58) | 0.76 (0.24-2.44) | 1.23 (0.44-3.47) | 1.46 (0.52-4.09) |
| Octogenarian | 1 ref | 2.82 (1.47-5.40) ** | 3.89 (1.98-7.66) *** | 1.53 (0.86-2.72) | 2.95 (1.70-5.09) *** | 3.92 (2.22-6.93) *** | 0.94 (0.47-1.86) | 2.57 (1.43-4.63) ** | 3.49 (1.97-6.20) *** |
| Nonagenarian | 1 ref | 1.47 (0.85-2.54) | 3.47 (1.67-7.18) | 0.76 (0.48-1.22) | 1.71 (1.09-2.70) * | 2.98 (1.82-4.90) *** | 0.79 (0.45-1.41) | 1.40 (0.81-2.42) | 2.72 (1.55-4.78) *** |
| **Socially engaged** |  |  |  |  |  |  |  |  |  |
| Younger elderly | 1 ref | 2.05 (1.25-3.37) ** | 1.90 (1.34-2.69) *** | 1.34 (0.83-2.17) | 1.44 (0.65-3.19) | 1.77 (1.09-2.88) * | 0.97 (0.70-1.36) | 1.10 (0.66-1.83) | 1.68 (1.27-2.21) *** |
| Octogenarian | 1 ref | 1.95 (1.33-2.86) ** | 2.12 (1.60-2.82) *** | 0.96 (0.63-1.46) | 1.16 (0.54-2.50) | 1.82 (1.09-3.03) * | 0.82 (0.62-1.09) | 1.34 (0.80-2.23) | 1.57 (1.18-2.08) ** |
| Nonagenarian | 1 ref | 2.60 (1.52-4.43) *** | 1.93 (1.29-2.88) ** | 0.66 (0.36-1.24) | 0.73 (0.10-5.33) | 1.95 (0.85-4.47) | 1.07 (0.75-1.52) | 1.98 (0.90-4.37) | 2.42 (1.56-3.78) *** |

*p<0.05 **p<0.01 ***p<0.001

Adjusted for age (continuous), gender, residence, years of schooling, marital status, economic status, living pattern, number of people living with, tobacco smoking, alcohol drinking, regular exercise, ADL, IADL, chronic disease (hypertension, diabetes, heart disease, stroke or CVD, cataract, digestive system disease, arthritis, Parkinson’s disease)

Supplementary Table S4. The association between cognitively stimulating, physically active/demanding, and socially engaged LAE change patterns and MCI reversion in sex subgroups.

|  | Low-low | Low-medium | Low-high | Medium-low | Medium-medium | Medium-high | High-low | High-medium | High-high |
| --- | --- | --- | --- | --- | --- | --- | --- | --- | --- |
| **Cognitively stimulating** |  |  |  |  |  |  |  |  |  |
| Male | 1 ref | 1.62 (1.04-2.52) * | 2.29 (1.14-4.55) * | 0.68 (0.43-1.07) | 1.71 (1.16-2.52) ** | 1.93 (1.19-3.12) ** | 0.75 (0.34-1.64) | 1.57 (0.91-2.73) | 2.25 (1.37-3.69) ** |
| Female | 1 ref | 1.94 (1.49-2.53) *** | 2.45 (1.22-4.89) * | 0.93 (0.71-1.22) | 1.90 (1.50-2.40) *** | 2.49 (1.45-4.27) ** | 0.66 (0.21-2.09) | 1.64 (0.82-3.30) | 3.28 (1.73-6.23) *** |
| **Physically active/demanding** |  |  |  |  |  |  |  |  |  |
| Male | 1 ref | 1.48 (0.81-2.71) | 2.32 (1.22-4.40) * | 0.73 (0.43-1.25) | 1.51 (0.92-2.46) | 1.95 (1.17-3.24) * | 0.82 (0.43-1.55) | 1.25 (0.72-2.19) | 1.96 (1.16-3.31) * |
| Female | 1 ref | 2.13 (1.29-3.52) ** | 4.32 (2.44-7.65) *** | 1.10 (0.71-1.69) | 2.60 (1.73-3.85) *** | 3.02 (1.98-4.60) *** | 0.89 (0.54-1.46) | 2.37 (1.56-3.60) *** | 3.10 (2.05-4.70) *** |
| **Socially engaged** |  |  |  |  |  |  |  |  |  |
| Male | 1 ref | 1.99 (1.32-3.01) ** | 1.92 (1.40-2.65) | 0.79 (0.48-1.31) | 1.21 (0.56-2.65) | 1.84 (1.09-3.09) * | 1.20 (0.88-1.63) | 1.49 (0.94-2.35) | 1.81 (1.35-2.42) *** |
| Female | 1 ref | 2.14 (1.53-3.01) *** | 2.07 (1.63-2.62) *** | 1.07 (0.77-1.49) | 1.17 (0.57-2.38) | 1.73 (1.16-2.58) ** | 0.85 (0.68-1.07) | 1.22 (0.76-1.96) | 1.60 (1.29-1.99) *** |

*p<0.05 **p<0.01 ***p<0.001

Adjusted for age (continuous), gender, residence, years of schooling, marital status, economic status, living pattern, number of people living with, tobacco smoking, alcohol drinking, regular exercise, ADL, IADL, chronic disease (hypertension, diabetes, heart disease, stroke or CVD, cataract, digestive system disease, arthritis, Parkinson’s disease)

Supplementary Table S5. The associations between overall, cognitively stimulating, physically active/demanding, and socially engaged LAE change patterns and MCI reversion when setting MMSE change≥4 points as MCI reverted status.

|  | Low-low | Low-medium | Low-high | Medium-low | Medium-medium | Medium-high | High-low | High-medium | High-high |
| --- | --- | --- | --- | --- | --- | --- | --- | --- | --- |
| Overall | 1 ref | 2.42 (1.64-3.58) *** | 3.57 (2.48-5.14) *** | 0.91 (0.63-1.30) | 2.81 (2.02-3.90) *** | 3.33 (2.41-4.60) *** | 1.08 (0.75-1.56) | 2.36 (1.69-3.32) *** | 3.44 (2.52-4.70) *** |
| Cognitively stimulating | 1 ref | 2.01 (1.54-2.63) *** | 2.78 (1.68-4.61) *** | 0.90 (0.68-1.18) | 2.17 (1.72-2.75) *** | 2.49 (1.74-3.58) *** | 0.94 (0.50-1.77) | 2.07 (1.35-3.18) ** | 3.07 (2.07-4.55) *** |
| Physically active/demanding | 1 ref | 2.10 (1.38-3.18) ** | 3.83 (2.41-6.09) *** | 1.00 (0.69-1.47) | 2.38 (1.68-3.38) *** | 3.05 (2.12-4.37) *** | 0.93 (0.60-1.46) | 2.23 (1.53-3.24) *** | 3.15 (2.19-4.53) *** |
| Socially engaged | 1 ref | 2.24 (1.68-2.98) *** | 2.06 (1.67-2.54) *** | 0.99 (0.73-1.34) | 1.42 (0.81-2.49) | 1.74 (1.20-2.53) ** | 0.95 (0.78-1.16) | 1.28 (0.89-1.83) | 1.76 (1.45-2.14) *** |

*p<0.05 **p<0.01 ***p<0.001

Adjusted for age (continuous), gender, residence, years of schooling, marital status, economic status, living pattern, number of people living with, tobacco smoking, alcohol drinking, regular exercise, ADL, IADL, chronic disease (hypertension, diabetes, heart disease, stroke or CVD, cataract, digestive system disease, arthritis, Parkinson’s disease)

Supplementary Table S6. The associations between all variables and MCI reversion after adding baseline MMSE score as a covariate.

| Variables | HR (95% CI) | P value |
| --- | --- | --- |
| **Age in years** | 0.98 (0.97-0.99) | <0.001*** |
| **Sex** |  | 0.018* |
| Female | 1 ref |  |
| Male | 1.22 (1.03-1.43) |  |
| Years of schooling | 1.01 (0.999-1.02) | 0.068 |
| **Location of residence** |  |  |
| Rural | 1 ref |  |
| Urban | 1.03 (0.94-1.14) | 0.498 |
| **Marital status** |  | 0.212 |
| Divorced/widowed /never | 1 ref |  |
| Married | 1.10 (0.94-1.31) |  |
| **Economic status** |  | 0.382 |
| Unfavorable | 1 ref |  |
| Favorable | 1.08 (0.91-1.26) |  |
| **Number of people living with** | 0.99 (0.96-1.03) | 0.693 |
| **Living pattern** |  | 0.632 |
| Alone or at nursing home | 1 ref |  |
| Living with family members | 1.62 (0.22-11.76) |  |
| **ADL** | 0.99 (0.92-1.07) | 0.792 |
| **IADL** | 0.99 (0.98-1.02) | 0.584 |
| **Smoke at present** |  | 0.444 |
| No | 1 ref |  |
| Yes | 1.07 (0.90-1.28) |  |
| **Drink at present** |  | 0.412 |
| No | 1 ref |  |
| Yes | 0.93 (0.79-1.10) |  |
| **Exercise at present** |  | 0.493 |
| No | 1 ref |  |
| Yes | 0.95 (0.82-1.10) |  |
| **Hypertension** |  | 0.212 |
| No | 1 ref |  |
| Yes | 1.11 (0.94-1.31) |  |
| **Diabetes** |  | 0.287 |
| No | 1 ref |  |
| Yes | 1.26 (0.82-1.92) |  |
| **Heart disease** |  | 0.342 |
| No |  |  |
| Yes | 1.13 (0.88-1.46) |  |
| **Stroke or CVD** |  | 0.078 |
| No | 1 ref |  |
| Yes | 0.72 (0.50-1.04) |  |
| **Cataract** |  | 0.894 |
| No | 1 ref |  |
| Yes | 0.98 (0.78-1.24) |  |
| **Digestive system diseases** |  | 0.166 |
| No | 1 ref |  |
| Yes | 1.22 (0.92-1.62) |  |
| **Arthritis** |  | 0.454 |
| No | 1 ref |  |
| Yes | 1.07 (0.90-1.25) |  |
| **Parkinson’s disease** |  | 0.279 |
| No | 1 ref |  |
| Yes | 1.74 (0.64-4.74) |  |
| **Baseline MMSE score** | 0.98 (0.94-1.02) | 0.382 |
| **LAE change pattern** |  |  |
| Low-low | 1 ref |  |
| Low-medium | 2.42 (1.64-3.58) | <0.001*** |
| Low-high | 3.58 (2.49-5.15) | <0.001*** |
| Medium-low | 0.91 (0.63-1.31) | 0.608 |
| Medium-medium | 2.80 (2.02-3.89) | <0.001*** |
| Medium-high | 3.34 (2.42-4.62) | <0.001*** |
| High-low | 1.09 (0.76-1.57) | 0.644 |
| High-medium | 2.36 (1.68-3.31) | <0.001*** |
| High-high | 3.46 (2.54-4.73) | <0.001*** |

*p<0.05 **p<0.01 ***p<0.001

Supplementary Table S7. The associations between overall, cognitively stimulating, physically active/demanding, and socially engaged LAE change patterns and MCI reversion when exclude participants with IADL disability at baseline.

|  | Low-low | Low-medium | Low-high | Medium-low | Medium-medium | Medium-high | High-low | High-medium | High-high |
| --- | --- | --- | --- | --- | --- | --- | --- | --- | --- |
| Overall | 1 ref | 2.16 (1.11-4.21) * | 2.78 (1.49-5.19) ** | 0.69 (0.37-1.30) | 2.12 (1.22-3.70) ** | 2.66 (1.55-4.55) *** | 0.93 (0.52-1.68) | 1.76 (1.02-3.02) * | 2.84 (1.69-4.78) *** |
| Cognitively stimulating | 1 ref | 1.74 (1.18-2.57) ** | 2.58 (1.33-5.01) ** | 0.76 (0.51-1.13) | 1.95 (1.38-2.75) *** | 2.35 (1.45-3.80) *** | 0.99 (0.46-2.14) | 1.81 (1.05-3.12) * | 2.66 (1.59-4.45) *** |
| Physically active/demanding | 1 ref | 1.96 (0.99-3.86) | 2.78 (1.39-5.57) ** | 0.57 (0.30-1.11) | 1.73 (0.97-3.08) | 1.93 (1.08-3.45) * | 0.78 (0.40-1.51) | 1.50 (0.83-2.70) | 2.16 (1.21-3.83) ** |
| Socially engaged | 1 ref | 1.91 (1.31-2.80) ** | 1.78 (1.32-2.41) *** | 1.02 (0.69-1.52) | 1.52 (0.84-2.77) | 1.59 (0.98-2.57) | 0.93 (0.72-1.21) | 1.16 (0.76-1.76) | 1.67 (1.31-2.13) *** |

*p<0.05 **p<0.01 ***p<0.001

Adjusted for age (continuous), gender, residence, years of schooling, marital status, economic status, living pattern, number of people living with, tobacco smoking, alcohol drinking, regular exercise, ADL, IADL, chronic disease (hypertension, diabetes, heart disease, stroke or CVD, cataract, digestive system disease, arthritis, Parkinson’s disease)
